# The complete chloroplast genome sequence of *Altingia yunnanensis*

**DOI:** 10.1080/23802359.2020.1721369

**Published:** 2020-02-03

**Authors:** Qiong Qiu, Dejun Yang, Linhong Xu, Yumei Xu, Yi Wang

**Affiliations:** a Institute of tropical forestry, Yunnan Academy of Forestry, Puwen, Yunnan, People’s Republic of China;; b The Key Laboratory of Rare and Endangered Forest Plants of State Forestry Administration, Kunming, Yunnan, People’s Republic of China

**Keywords:** *Altingia yunnanensis*, chloroplast, Illumina sequencing, phylogenetic analysis

## Abstract

The first complete chloroplast genome (cpDNA) sequence of *Altingia yunnanensis* was determined from Illumina HiSeq pair-end sequencing data in this study. The cpDNA is 160,860 bp in length, contains a large single-copy region (LSC) of 89,162 bp and a small single-copy region (SSC) of 19,008 bp, which were separated by a pair of inverted repeat (IR) regions of 26,325 bp each. The genome contains 130 genes, including 85 protein-coding genes, 8 ribosomal RNA genes, and 37 transfer RNA genes. Further, the phylogenomic analysis showed that *A. yunnanensis* and *Altingia excelsa* clustered in a clade in Saxifragales order.

*Altingia yunnanensis* is the species of the genus *Altingia* within the family Altingiaceae. It distributes in the southeast of Yunnan (Yang et al. 1996). This species is of great significance for studying the taxonomic status of *Altingia* and Hamamelidaceae (Ickert-Bond et al. 2007). *Altingia yunnanensis* is also constructive species in the tropical rain forest of China (Xu et al. 2004). However, there have been no genomic studies on *A. yunnanensis.*

Herein, we reported and characterized the complete *A. yunnanensis* plastid genome. The GenBank accession number is MN106248. One *A. yunnanensis* individual (specimen number: 201807060) was collected from Puwen, Yunnan Province of China (22°25′25′′N, 101°6′31′′E). The specimen is stored at Yunnan Academy of Forestry Herbarium, Kunming, China and the accession number is YAFH0012862. DNA was extracted from its fresh leaves using DNA Plantzol Reagent (Invitrogen, Carlsbad, CA).

Paired-end reads were sequenced by using Illumina HiSeq system (Illumina, San Diego, CA). In total, about 24.5 million high-quality clean reads were generated with adaptors trimmed. Aligning, assembly, and annotation were conducted by CLC de novo assembler (CLC Bio, Aarhus, Denmark), BLAST, GeSeq (Tillich et al. 2017), and GENEIOUS v11.0.5 (Biomatters Ltd., Auckland, New Zealand). To confirm the phylogenetic position of *A. yunnanensis*, the other 13 species of order *Saxifragales* from NCBI were aligned using MAFFT v.7 (Katoh and Standley 2013). The Auto algorithm in the MAFFT alignment software was used to align the 16 complete genome sequences and the G-INS-i algorithm was used to align the partial complex sequences. The maximum likelihood (ML) bootstrap analysis was conducted using RAxML (Stamatakis 2006); bootstrap probability values were calculated from 1000 replicates. *Chloranthus spicatus* (EF380352) and *Buxus microphylla* (EF380351) were served as the out-group.

The complete *A. yunnanensis* plastid genome is a circular DNA molecule with the length of 160,860 bp, contains a large single-copy region (LSC) of 89,162 bp and a small single-copy region (SSC) of 19,008 bp, which were separated by a pair of inverted repeat (IR) regions of 26,325 bp each. The overall GC content of the whole genome is 37.9%, and the corresponding values of the LSC, SSC, and IR regions are 36.0, 32.2, and 43.1%, respectively. The plastid genome contained 130 genes, including 85 protein-coding genes, 8 ribosomal RNA genes, and 37 transfer RNA genes. Phylogenetic analysis showed that *A. yunnanensis* and *Altingia excelsa* clustered in a unique clade in Saxifragales order (Figure 1). The determination of the complete plastid genome sequences provided new molecular data to illuminate the order Saxifragales evolution.

**Figure 1.**
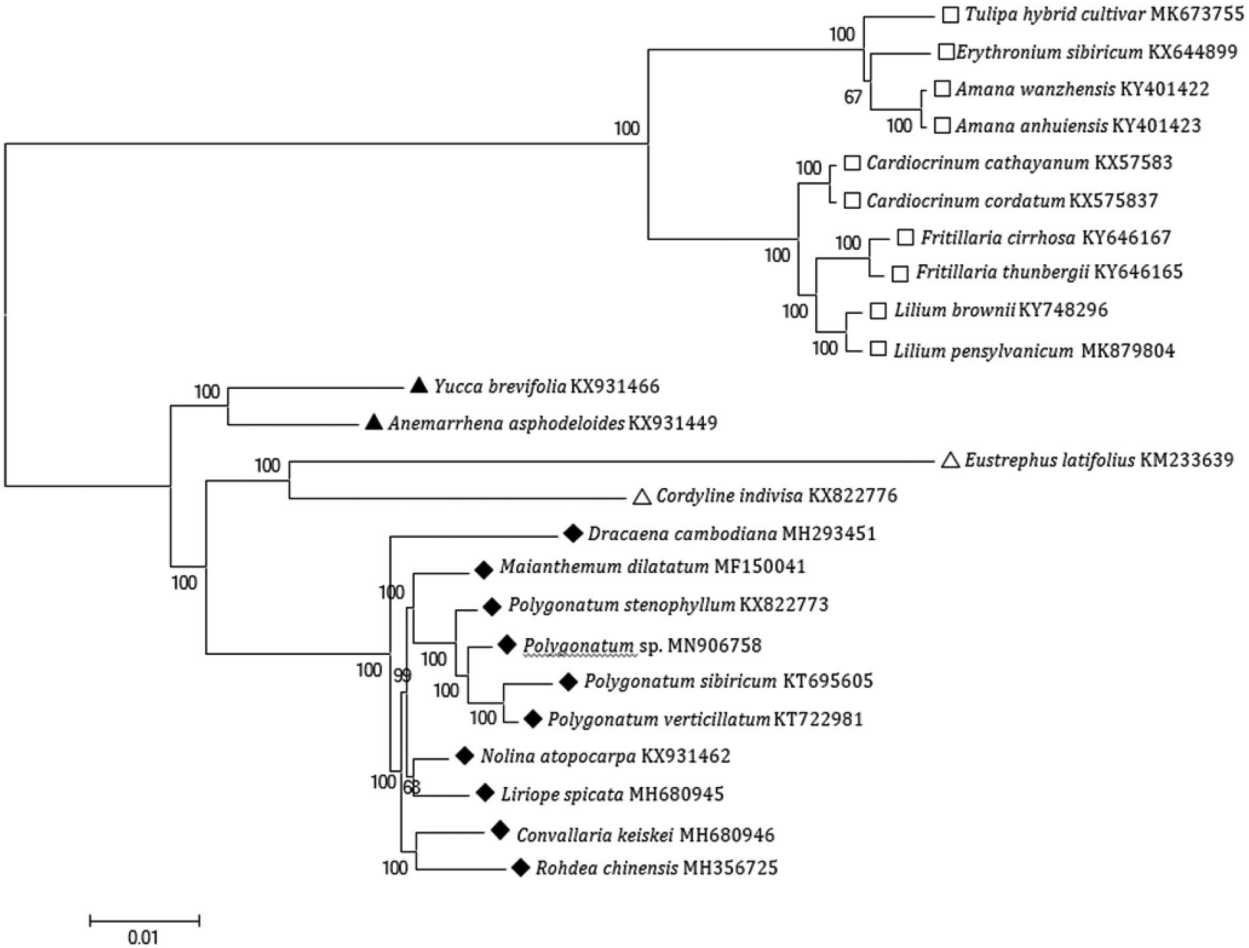
The maximum-likelihood tree based on the fourteen chloroplast genomes of order *Saxifragales*. The bootstrap value based on 1000 replicates is shown on each node.
